# Relevance of Trust Marks and CE Labels in German-Language Store Descriptions of Health Apps: Analysis

**DOI:** 10.2196/10394

**Published:** 2018-04-25

**Authors:** Urs-Vito Albrecht, Uta Hillebrand, Ute von Jan

**Affiliations:** ^1^ Peter L Reichertz Institute for Medical Informatics Hannover Medical School Hannover Germany

**Keywords:** mobile phone, mobile health app, quality assessment, quality seals, medical device regulation

## Abstract

**Background:**

In addition to mandatory CE marking (“CE” representing *Conformité Européenne*, with the CE marking being a symbol of free marketability in the European Economic Area) for medical devices, there are various seals, initiatives, action groups, etc, in the health app context. However, whether manufacturers use them to distinguish their apps and attach relevance to them is unclear.

**Objective:**

The objective was to take a snapshot of quality seals, regulatory marks, and other orientation aids available on the German app market and to determine whether manufacturers deem such labels relevant enough to apply them to their apps, namely as reflected by mentions in app description texts in a typical app store (ie, Apple’s App Store).

**Methods:**

A full survey of the metadata of 103,046 apps from Apple’s German App Store in the Medicine and Health & Fitness categories was carried out. For apps with German-language store descriptions (N=8767), these were automatically searched for the occurrence of relevant keywords and validated manually (N=41). In addition, the websites of various app seal providers were checked for assigned seals.

**Results:**

Few manufacturers referenced seals in the descriptions (5/41), although this would have been expected more often based on the seals we were able to identify from the seal providers’ Web pages, and there were 34 of 41 that mentioned CE status in the descriptions. Two apps referenced an app directory curated by experts; however, this is not an alternative to CE marks and seals of approval.

**Conclusions:**

Currently, quality seals seem to be irrelevant for manufacturers. In line with regulatory requirements, mentions of medical device status are more frequent; however, neither characteristic is effective for identifying high-quality apps. To improve this situation, a possibly legally obligatory, standardized reporting system should be implemented.

## Introduction

Calls for clear labels reflecting “quality” aspects of health-related apps are becoming ever louder in the medical community [1]. Such labels are intended to provide interested laypersons as well as professionals with a quick and easy way to identify apps with an adequate level of quality for use on their mobile phones or tablets, which is not an easy task considering the highly dynamic, borderless, and largely unregulated nature of the app market [2-4]. The demand is justified; often, it is a challenge for users to make a decision to use an app or to refrain from doing so because provided information is limited in many cases. Although it is already difficult to identify an app having the desired features among the often large variety of those available, assessing whether an app is a high-quality product poses an even greater challenge. For health experts, content-related assessments are often unproblematic, but when technical aspects, data protection, and data security come into play, they are often out of their depth as well. Carrying out such assessments in an adequate manner requires not only knowledge and experience, but is often time consuming and technically demanding. If reliable, seals of approval and other marks of quality have the potential to simplify the situation for users because they hold the promise of an in-depth (quality-related) inspection by independent third parties that users can base their decisions on. Driven by these demands, quality seals are increasingly being offered and a new business field appears to be establishing itself. Various commercial and institutional initiatives are trying to meet the demand.

This paper is dedicated to the question of whether quality seals, regulatory marks, and other orientation aids are available on the market and, if so, which ones are available and do they (and how often) appear in the app context and to what extent are they being used in the app description texts of a typical app store (ie, Apple’s App Store). Subsequently, the relevance of quality seals and CE markings (“CE” representing *Conformité Européenne*, with the CE marking being a symbol of free marketability in the European Economic Area) will be discussed on the basis of the results. For the initial assessment of the situation, we focused on apps listed in Apple’s German storefront as well as quality seals relevant to the German or European market.

## Methods

### Overview

To answer the aforementioned questions, a two-stage process was used. First, relevant quality seals and corresponding keywords were identified. These were then applied to the metadata of the full set of apps listed within the Health & Fitness as well as Medicine categories of Apple’s App Store on a specific date to determine whether and how often manufacturers reference quality labels within the information they provide. The composition and frequency of the quality labeling of apps is then described to what degree they are actually used.

### Search Strategy for Identifying App Seals

Official test institutions specializing in health-related apps and their specific requirements, standardized and (universally) accepted testing procedures, or registers that list various approaches applicable in a health context are rare. Ultimately, this is not surprising because regulations—and, therefore, obligatory testing of quality- and transparency-related aspects—only apply to a limited subset of apps on the market. For unregulated apps, a number of private institutions and initiatives try to step in by providing various quality marks and orientation guidelines, but similar to the seemingly chaotic manner in which apps are presented in the stores, potential approaches—and the quality of their evaluation processes—are equally confusing for end users.

Our assessment of seals, orientation guidelines, or similar approaches mentioned by manufacturers was based on relevant key terms we found based on searches within the literature (PubMed, IEEE, and Scopus) as well as other online media (internet). In addition, we also included our own previous work to identify relevant key terms [5,6].

### Acquisition of Apps Listed Within the Health & Fitness and Medicine Categories

In the first step, an initial list of apps—or, to be more precise, their names and numeric IDs—listed in two categories, namely Medicine and Health & Fitness of the German Apple App Store, was acquired using R-based scripts [7] by parsing the Web pages for both categories. Using these scripts, on February 5, 2018, it was possible to read information (app names and unique identifiers) for 103,046 apps listed in the two categories. The readout of the corresponding meta-information was done in a second step and took place between February 5, 2018 and February 6, 2018. Again, R-based scripts, this time using the iTunes search application programming interface provided by the App Store provider were used to retrieve the meta-information for the initially acquired app list based on the previously acquired unique identifiers. The acquired data were stored in an SQLite-based database for further analysis.

### Semiautomatic Retrospective App Store Analysis

For analyzing the data acquired for the apps of the two target categories, a newly developed method for semiautomatic retrospective App Store analysis (“SARASA” for future reference) was used (details are to be published soon). The method provides a step-by-step filtering of apps by formal criteria. In addition to keyword searches (with the use of Boolean operators where desired), it also allows for differentiation by factors deduced from the original information (eg, automatically determined language and text complexity of app store descriptions, topic analyses). Using the semiautomatic retrospective App Store analysis, the intermediate and final results of the filter process can be presented in descriptive form and, if desired, in a graphical manner as well. At the end of an analysis according to the semiautomatic retrospective App Store analysis filter scheme, there is a selection of apps that can then be manually validated. For data used in this case, this evaluation was performed by the authors based on further formal criteria. There was only a single initial disagreement relating to one app that ambiguously stated conformity to medical device regulation in its store description. This app was finally found not to be a medical device itself, but rather to have the sole purpose of providing information about a medical device to be used in the context of fertility tracking. The problem was easily resolved based on the facts determined using additional Web-based searches (Google) and the app was excluded from further analysis.

### Formal Inclusion Criteria

**Figure 1:**
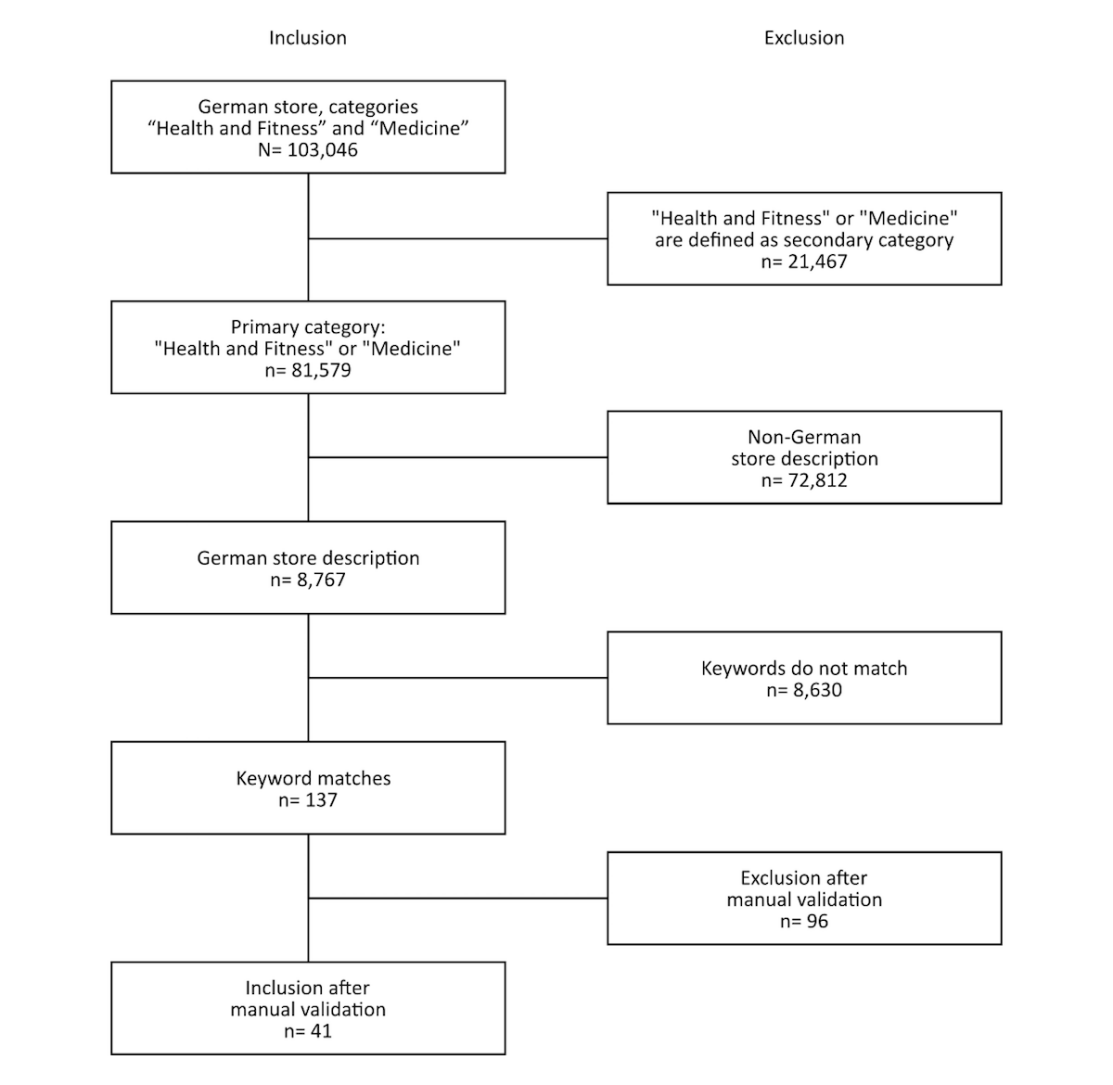
Flowchart of the inclusion and exclusion of apps acquired on February 5, 2018. N=41 apps were finally available.

Only the apps from Apple’s German App Store, whose primary category was Medicine or Health & Fitness, and whose descriptions were written in German and contained predefined keywords (see Textbox 1) were included in the study; apps with store descriptions in languages other than German were not included. This may have caused exclusion of some multilingual apps with a German interface that were missing a translation in the store description. Apps that were only assigned to the two categories via their secondary category were not included in this analysis (Figure 1).

**Keywords used to identify matching apps via their store descriptions, stratified by “labeled medical device,” “seal of approval / quality seal,” and “other.”**

**Labeled medical device**

Keywords: Medizinprodukt; medical device; CE-; CE mark; CE label; 93/42/; 2017/745; Medical device directive; MDD; Medical Device Regulation; MDR

**Seal of approval / quality seal**

Keywords: Siegel; Prüfzeichen; Ehrenkodex; Trusted App; AppCheck; Qualitätsprodukt Internetmedizin; Qualität durch Transparenz; Geprüfte App; EuroPrisSe; Privacy Seal; mWellth-Certificate; SocialWellth; eprivacy; Health On the Net; HealthOn; mediatest digital; TÜV; DiaDigital; Diabetes-App-Siegel; Zentrum für Telematik und Telemedizin GmbH; ZTG; Stiftung Warentest; Bundesverband Internetmedizin; BIM; aktionsforum gesundheitsinformationssystem; afgis; Happtique; tekit Consult Bonn; mWellth; app-quality.com; Appquality-Alliance

**Other**

Keywords: Kodex; Privacy Code of Conduct; HONcode; HON-code; Quality Alliance; AQUA; Medical App Journal; JMIR mHealth peer review; Journal of Medical Internet Research; JMIR; JMU; iMedicalApps.com; MyHealthApps; European Directory of Health Apps; App Script; NHS Health Apps Library; Myhealthapps.net; App Chronic Disease Checklist; ACDC; Mobile Application Rating Scale; MARS; User Version of Mobile Application Rating Scale; uMARS; ClassifyDroid; Mobile Apps Assessment and Analysis System; MARS; interactive Mobile App Review Toolkit; IMART; App-Synops; Zertifikat; BfDI

### Search Strategy for Identifying Matches Within the Store Descriptions

Of the 103,046 apps with German-language description texts (N=8767, with the language used within the texts automatically identified using the cld2 package in R, which provides an interface to Google’s C++-based “Compact Language Detector 2” library), the keywords listed in Textbox 1 were used to identify potentially relevant apps. To not miss out on any possible matches, a case-insensitive search process was applied for defined terms or spelled-out names of initiatives, seals, orientation guidelines, checklists, or similar. However, case sensitivity was required for matching acronyms. The description texts of the apps identified in this manner were then manually checked for plausibility. For example, apps that explicitly stated that they are “not a medical device” or used any other phrasing within their descriptions precluding use as a medical device were marked separately.

**Table 1:** Types and numbers of seals identified from the Web search (stratified by primary category).

Seal of approval / quality seal	Provider	Labels/seals assigned to health-related apps as specified on the providers’ Web pages (N=100)	Corresponding apps found within the store (N=52)	App category		
				H&F^a^ (n=10)	Medicine (n=31)	H&F and Medicine (n=41)
AppCheck [8]	Zentrum für Telematik und Telemedizin GmbH; ZTG	36	15	0	0	0
CE-Kennzeichnung / CE-mark (various databases, the German DB can be found in [9])	Responsible notified body	39	20	7^b^	27^c^	34
CheckYourApp [10]	MIASEC GmbH	Seals not listed, but can be researched by app name.	N/A^d^	0	0	0
Diabetes-App-Siegel [11]	DiaDigital der AG Diabetes und Technologie der Deutschen Diabetes Gesellschaft e.V.	5	5	0	0	0
eprivacyseal; ePrivacyApp [12]	ePrivacy GmbH	5	4	1	0	1
EuroPriSe European Privacy Seal [13]	EuroPrisSe GmbH	Apps not listed separately	N/A	0	0	0
HealthOn-Siegel [14]	HealthOn	11	6	0	2	2
HONcode [15]	Health On the Net Foundation	Unpublished	N/A	0	0	0
Test [16]	Stiftung Warentest	Apps not listed separately but as parts of various test reports	N/A	1	0	1
Trusted App [17]	mediaTestdigital GmbH	4	2	1	0	1
TÜV SÜD Software-Prüfzeichen [18]	TÜV Süd Produkt Service GmbH	Unpublished	N/A	0	0	0
Qualität durch Transparenz; afgis-Qualitätslogo [19]	aktionsforum gesundheitsinformationssystem afgis e.V.	Unpublished	N/A	0	0	0
Qualitätsprodukt Internetmedizin [20]	Bundesverband Internetmedizin BIM e.V.	Unpublished	N/A	0	0	0
Other	Other	Unclear	N/A	0	2	2

^a^Health and Fitness.

^b^For 6 of 7 CE-marked Health & Fitness apps, we were able to confirm class I either from statements by the manufacturers themselves or from information listed in the DIMDI (Deutsches Institut für Medizinische Dokumentation und Information [German Institute of Medical Documentation and Information]) database. For the remaining app, we were unable to determine its class, but suspect it to belong to class I as well (due to its functionality).

^c^Of the 27 CE-marked apps listed in the Medicine category, 17 apps were specified as class I, 1 app as class Im, 6 apps as class IIa, 2 apps as class IIb, and for one app, we were unable to determine its class, but again suspect it to be of class I due to its functionality.

^d^Not available or not applicable.

### Identification of Apps With Definite Seal Assignments

As an additional validation step, we also checked for a relationship between the seals actually awarded by various initiatives active in this business and their actual mention by manufacturers within the App Store descriptions. This was intended to counteract a potential bias caused by apps that are mentioned by the initiatives, but are either not (or no longer) listed in the App Store or simply fail to mention the respective seal within their descriptions. For this purpose, the publicly available app information provided by the initiatives/providers (namely app and manufacturer names as listed on the initiatives’ Web pages on February 7, 2018) was used and apps identified in this manner were deemed as having their “seal status” validated (Table 1). The acquired information included names, providers, and URLs of the labels or seals, as well as the number of apps listed. Based on the store descriptions, the number of apps that stated to be using the respective seal or label were recorded as well.

## Results

There were a number of quality marks and initiatives that deal with the testing and evaluation of apps and general health information. We counted 13 individual seals in our search, and the organizational character (eg, public or private sector, corporate form) of those providing these seals varied greatly (Table 1). There were also various differences in the objectives and methods of the offers, which have been described in detail elsewhere [6,21] and are therefore not the subject of this work. These mostly related to what the evaluation process focused on (eg, content validation, technical aspects, and information security/data protection as well as usability or any combinations thereof), but differences were also attributable to how those initiating the evaluation were organized or financed (eg, private initiatives, patient organizations, commercial offers) and who was recruited for performing the evaluation (eg, patients/laypersons, medical or technical experts).

There was little evidence that the manufacturers and vendors made use of references to any seals or quality-related marks and reported on them in the descriptions they provided for their apps. In total, only for 41 of 8767 apps (all German-language apps with their primary category being either Health & Fitness or Medicine; Figure 1) were there any mentions of any type of quality label (0.47%). The largest proportion of these (34/41) were apps with medical device status (CE mark). Of these 34, seven were in the Health & Fitness category and 27 in the Medicine category. Only five of 41 had been awarded nongovernmental quality seals or labels, three in the Health & Fitness category and two in the Medicine category. For two of 41 apps, there was a reference to an app directory curated by experts. We found no apps that were labeled as medical devices and also carried a seal of approval. For four apps found in the Medicine category as well as four additional apps in the Health & Fitness category, medical device status was expressly excluded.

With respect to app demographics (Table 2), the 41 apps significantly differed from the total group of apps with German descriptions with respect to the time that had passed since the last update (Wilcoxon rank sum test for unpaired samples, two-sided, confidence level 95%, *U*=237,440, *P*<.001) and in the length of their store descriptions (*U*=84,360, *P*<.001). However, for overall age, app size, price, and star ratings, differences were insignificant. A more in-depth analysis of other app characteristics associated with assigning a quality label will be part of further work and will be discussed in detail then. To ascertain that we did not inadvertently miss any apps by restricting our app selection to apps listed with primary categories of either Health & Fitness or Medicine, the keyword-based search was also applied to the other apps with a German description. However, there was only one Health & Fitness app (primary category “Sport”) that mentioned a medical device certification and another one that explicitly excluded medical device status. There were no additional mentions of relevant seals or similar markings.

The surprisingly low number of seals mentioned in our data led us to consider checking the relationship between seals officially awarded by the initiatives (as designated by information on the websites of the respective seal providers) and their actual mention by manufactures in the App Store descriptions. This was done to rule out that our keywords were insufficient to find seals that were assigned. However, this concern was unfounded, as out of 100 apps we identified as being officially allowed to use a seal based on the information published by the seal providers, there were still only 41 matches also found on the App Store, even when allowing for slightly different spellings (eg, missing whitespaces, differing capitalization) of the names of the manufacturer and apps (41%, 41/100; Table 1). In addition to apps sometimes being withdrawn from the App Store for no obvious reasons, this may also be due to apps being renamed, takeovers of manufacturers by other companies and subsequent retraction of the apps from the store, etc. Evidently, such events are either not appropriately communicated to the initiatives that assigned a specific seal to the respective app or the initiatives themselves fail to update their register of apps carrying that specific seal on a regular basis (eg, every half year or annually), resulting in apps that are no longer available on the market still being included.

In our evaluation, we made sure that the apps we identified on the Web pages of the initiatives were listed on Apple’s App Store or additionally on Google’s Play Store. Only slightly more than one-third of the “seal carrying apps” identified from the Web pages of the seal providing initiatives could be found on Apple’s App Store. The identified apps were accepted as a sample of apps “assuredly carrying a seal or other quality mark,” which is by no means reflected in the app descriptions. As mentioned previously, only for 41 of 8767 (0.47%) apps were there any references to one of the previously identified seals within the app descriptions listed on Apple’s App Store.

**Table 2:** App demographics for apps with a primary category of either Medicine or Health &amp; Fitness and German-language store descriptions (N=8767) as well as for the manually validated (N=41) apps.

App demographic	All apps with German-language store descriptions and primary category Medicine or Health & Fitness (N=8768), median (IQR)	Manually validated apps (N=41), median (IQR)	*U* value^a^	*P* value
Overall age (months)	30.74 (33.21)	28.14 (32.84)	N/A^b^	N/A
Time since last update (months)	9.48 (20.68)	3.62 (12.92)	237,440	<.001
Size (MB)	28.08 (37.57)	30.29 (46.22)	N/A	N/A
Price (€; paid apps only)	2.29 (2.5)^c^ for n=1706 paid apps	3 paid apps (5.99, 43.99, and 69.99)	N/A	N/A
Length of description (characters)	922 (1419.5)	2352 (1610)	84,360	<.001
Average rating (current version; stars)	4.5 (2) for n=2841 rated apps	4 (1.75) for n=26 rated apps	N/A	N/A
Average rating (all versions; stars)	4 (2) for n=3287 rated apps	4 (1) for n=27 rated apps	N/A	N/A

^a^Wilcoxon rank sum test where applicable.

^b^N/A: not applicable. Differences between both groups were descriptively too small to warrant testing.

^c^Price range: €0.49 to €499.99.

Surprisingly, it was not the quality seals but the CE marking that was most often mentioned within the app descriptions (Table 1); for a total number of 34 apps, manufacturers declared their app to be a medical device. Based on a previously performed search in the databases of the German DIMDI (Deutsches Institut für Medizinische Dokumentation und Information [German Institute of Medical Documentation and Information], responsible for the official German register of medical products), we initially expected to find just 20 German-language apps with CE markings on the store. However, we found that the 14 additional apps we had identified via the information contained in the store descriptions were registered in other European countries and were therefore not part of the DIMDI dataset.

Our investigation also showed that the number of apps that were explicitly mentioned as not being a medical device exceeded the number of apps for which there was a reference to quality-related seals or labels. Medical device status was explicitly excluded eight times in total (which we believe to be valid based on the information given in the respective store descriptions), and there were only five references to seals or labels (Table 1). Two apps only referred to being listed on a website curated by experts, which although possibly being an alternative, may or may not provide users with the same level of confidence about quality as CE markings or established quality seals.

## Discussion

### Principal Findings

The aim of this study was to provide an up-to-date overview of how official regulatory markings for medical devices, namely the CE marking applicable in Europe, and marks supposedly designating quality (eg, quality seals) are used in apps found on the market. For this, we determined how often these are mentioned in German-language store descriptions of individual apps in the German storefront of Apple’s App Store, with primary categories being specified as either Health & Fitness or Medicine. This was done to investigate whether manufacturers prefer to publish apps that are subject to official regulation (due to their functionality) or whether they would rather limit themselves to apps that do not contain functions requiring adherence to medical device regulations and for which quality seals would therefore be sufficient. App characteristics were also factored in to narrow down potential reasons for either alternative being preferred.

The small number of health-related labels or seals being mentioned (5 of 8767 apps with a German description and primary category of Medicine or Health & Fitness, 0.06%) is somewhat astonishing because, apart from the mandatory CE marking for medical devices, which is required by regulation, these are low-threshold and relatively easy to apply options that may especially be relevant for nonmedical products and can be used for advertising purposes. However, manufacturers and providers seem to attach little importance to them. Reasons may be similar to those often given with respect to quality seals for websites, which are also used less frequently than expected. Here too, seals of approval are fairly unknown, although there are many different seals that are potentially applicable in this context. Wetter [22] suspects that it is this considerable number of seals (and the different approaches they stand for) as well as the resulting competition among seal-awarding authorities that confuses those who have to decide which seal to apply for. The decision whether to apply for a seal or certification or to pursue another strategy to convince users of a product's quality remains open [22]. Whether quality seals assigned to websites actually give an indication of quality is also controversial. As was already shown by Keselman et al in 2008 [23], there is not always a connection between defined quality criteria being fulfilled and websites providing accurate content, and the same probably holds true in an app context. Therefore, it would be understandable if, especially for apps that are deemed medical devices, manufacturers and providers were to concentrate on advertising CE markings rather than quality seals, presumably also because this is already deemed sufficient with respect to marketing considerations in this context.

In addition, for health apps, the concept of “quality” still needs to be discussed and there are several dimensions to consider. Beyond the perception of quality by users and how this perceived quality influences decisions to purchase and install apps within the secondary health care market, manufacturers are aware that obtaining a CE marking significantly increases (or is presumed to increase) chances of entering the primary (and insurer-paid) health care market. However, although CE markings are often regarded as a quality feature, one needs to remember that they are representations of manufacturers following regulatory requirements, which is the basis for being allowed to market medical products (market harmonization), rather than labels representing a product’s actual quality [24,25]. As a rule, a detailed quality check, which many users may expect, does not take place in this context. However, it can be stated that the conformity assessment procedure is at least transparent in its requirements and thus provides more information about what is to be expected than is the case with some providers of quality seals.

It may also be possible that the financial and time expenditure involved in obtaining a seal or other quality approval is an obstacle [26,27]. For apps for which this is a requirement, following regulation is just as time consuming and costly, and obtaining a seal is voluntary and may thus be foregone if manufacturers perceive no obvious benefit. Possibly, the fees (usually in the range of several hundred to several thousand Euros) often required for developers to be able to obtain these seals or approvals for their apps may be a deterrent as well; some are available for free, but others require either a one-time, monthly, or yearly fee or (paid) membership in an organization, which may be perceived as a continuing burden especially for apps with limited potential for commercial success. However, there are some measures that may hold much lower thresholds for manufacturers while aiding potential users in their decisions. To provide future users with low-threshold, cost-neutral support, it would be conceivable to produce meaningful descriptions following a standardized structure that covers relevant information (eg, about sources used for implementing the app, qualifications of those involved) rather than only providing marketing phrases. We already previously published drafts for such standardized app reporting [28,29], which would also cover CE markings, quality seals, or results of any other tests and/or studies possibly performed. This approach would provide users with relevant information they need to make purchase decisions and it would not be an undue burden—sometimes mentioned in the literature [30]—on developers because they should have the corresponding information at their fingertips. Making such standardized reporting obligatory (albeit without regularly performed mandatory checks by official bodies), similar to what is currently required with respect to an imprint [21,31-33], should certainly be discussed by all stakeholders. This would ensure that users are provided with relevant information before downloading. Even if some items were only listed as “information unavailable,” users would benefit from not having to invest too much effort to determine this.

### Limitations

Our study was subject to the following limitations. Our focus was specifically on the German-speaking region, reflected by the evaluation of apps for which German-language store descriptions had been provided. We also considered only quality initiatives and regulatory approaches relevant in the German-speaking regions, and we had to limit ourselves to app registrations found in the DIMDI database due to the fees required for searching in other regulatory databases. For the future, with the introduction and adoption of unique device identifiers and the European Database on Medical devices, as required under medical device regulation, it will henceforth become easier to assess all medical devices, including apps, placed on the European market. Ongoing work, to be published later, also considers regulatory requirements (eg, US Food and Drug Administration approval) as well as seals found internationally to determine whether there are significant differences between geographic regions. Also, there is currently no legal obligation to publish information about following regulation (eg, medical device related) within the store descriptions and because our evaluation was solely based on the information provided here, we may have missed some apps only mentioning adherence to regulation within the apps themselves and/or on related Web pages and manuals, which we did not evaluate.

Even though considerable effort was made to identify existing quality-related seals with a special focus on applicability in a health context, it cannot be ruled out that there are possible, probably recently established, initiatives we did not find. Also, the seal providers’ Web pages are often not conducive for identifying seals that have been assigned to apps. Of course, seal providers are also called on to not only publish information about what they offer but also to ensure that this information can be found using relevant search engines. They should also take care to keep their information about validated apps up to date.

In addition, we only used the description texts of the apps for our keyword-based searches. No statements can be made on the basis of this information about whether this actually reflects the information provided within the apps themselves or elsewhere. Another limitation of the work is its limitation to one single app store. Therefore, it remains to be seen whether our result can also be reproduced for other app stores (eg, Google’s Play Store).

### Conclusions

The prevalence of labels in App Store descriptions is negligible. Therefore, it is reasonable to assume that these are not of any noteworthy relevance for manufacturers when it comes to providing information and promoting their products. Only medical device designations are communicated regularly and in full, which does take account of the regulatory necessity, but also helps with differentiating them from other nonregulated and labeled products in terms of advertising effectiveness. To improve the quality-related information provided in the stores, a standardized reporting process used for compiling the app description text is recommended. A legal obligation to do so would contribute to the effective enforcement of appropriate information.

## Abbreviations

CE: Conformité Européenne
DIMDI: Deutsches Institut für Medizinische Dokumentation und Information [German Institute of Medical Documentation and Information]
